# Effect of achieved hemoglobin level on renal outcome in non-dialysis chronic kidney disease (CKD) patients receiving epoetin beta pegol: MIRcerA CLinical Evidence on Renal Survival in CKD patients with renal anemia (MIRACLE-CKD Study)

**DOI:** 10.1007/s10157-018-1649-0

**Published:** 2018-10-05

**Authors:** Terumasa Hayashi, Yukari Uemura, Michiko Kumagai, Masashi Kimpara, Hiroyuki Kanno, Yasuo Ohashi, MIRACLE-CKD Study Group

**Affiliations:** 1 Department of Kidney Disease and Hypertension, Osaka General Medical Center, 3-1-56 Bandai-higashi, Sumiyoshi-ku, Osaka, 558-8558 Japan; 2 Biostatistics Department, Central Coordinating Unit, Clinical Research Support Center, The University of Tokyo Hospital, Tokyo, Japan; 3 Pharmacovigilance Department, Chugai Pharmaceutical Co., Ltd., 2-1-1 Nihonbashi-Muromachi, Chuo-ku, Tokyo, 103-8324 Japan; 4 Department of Integrated Science and Engineering for Sustainable Society, Chuo University, Tokyo, Japan

**Keywords:** Anemia, Chronic kidney disease, Erythropoiesis-stimulating agents, Renal survival

## Abstract

**Background:**

Previous randomized-controlled trials have shown that targeting higher hemoglobin (Hb) levels using high dose of ESA in non-dialysis chronic kidney disease (NDCKD) patients resulted in poorer cardiovascular outcome; however, it remains unknown how high Hb levels achieved by ESA in clinical practice dose could affect renal outcome.

**Methods:**

In a multicenter prospective observational study, Japanese NDCKD patients with an estimated glomerular filtration rate (eGFR) of ≥ 6 mL/min/1.73 m^2^ and renal anemia (Hb < 11 g/dL) treated with epoetin beta pegol (C.E.R.A.) for the first time were divided into two groups by Hb level (< 11 g/dL or ≥ 11 g/dL) in Week 12 of C.E.R.A. treatment (Week 12 Hb). Renal outcome was defined as time until the first occurrence of one of the following: progression to renal replacement therapy, serum creatinine doubling, or eGFR falling below 6 mL/min/1.73 m^2^. The effect of Week 12 Hb on the onset of renal events was assessed by the Kaplan–Meier and multivariate Cox regression analyses.

**Results:**

In the landmark analysis which included 2851 patients, Kaplan–Meier renal survival rate was 37.57% in the < 11 g/dL group and was significantly higher (51.47%) in the ≥ 11 g/dL group (*P* < 0.0001). Multivariate Cox regression analysis revealed significantly higher risk of renal events in the < 11 g/dL group than in the ≥ 11 g/dL group (hazard ratio: 1.26; 95% confidence interval: 1.05–1.51; *P* = 0.0103).

**Conclusions:**

The results suggest that week 12 Hb levels ≥ 11 g/dL achieved with C.E.R.A. treatment were associated with better renal outcomes than Hb levels < 11 g/dL.

**Electronic supplementary material:**

The online version of this article (10.1007/s10157-018-1649-0) contains supplementary material, which is available to authorized users.

## Introduction

Anemia occurs at a relatively early stage in non-dialysis chronic kidney disease (NDCKD) patients [1], and is related to renal function outcome, cardiovascular comorbidities, and mortality [2]. Investigations of the effects of treating anemia with erythropoiesis-stimulating agents (ESAs) have had conflicting findings in terms of renal protection, inhibition of cardiovascular events, and improvement of life expectancy [3–8]. In addition, there have been no conclusions on the optimal hemoglobin (Hb) level for starting ESA therapy or the optimal target Hb level for maintenance ESA therapy. Gouva et al. compared a group with Hb maintained at 13 g/dL by starting ESA early and a group in which ESA therapy was started at Hb ≤ 9 g/dL; they reported a higher renal survival rate when ESA therapy was started early [3]. The JET-STREAM study conducted in Japan also found that the early initiation of anemia treatment when Hb levels decreased below 11 g/dL could be more effective at reducing the risk of renal events in NDCKD patients with anemia compared with the initiation of ESA therapy at an Hb level of 9 g/dL or even 10 g/dL [4].

There have also been reports related to target Hb levels in ESA therapy and outcomes in NDCKD patients from a series of large randomized-controlled trials (RCTs), including the CHOIR [5], CREATE [6], and TREAT [7] studies. In these studies, setting the target Hb level higher than 13 g/dL in NDCKD patients gave no benefit in preserving renal function but increased the incidence of cardiovascular and stroke events. In the CREATE study, more patients progressed to dialysis when the target Hb level was 13.5 g/dL than when it was 11.3 g/dL. Based on those results, the 2012 Kidney Disease: Improving Global Outcomes (KDIGO) Guidelines set an Hb target of 10–11.5 g/dL for NDCKD patients [9].

Nevertheless, secondary analyses of those RCTs drew attention to the impact of ESA hyporesponsiveness on outcome by showing that patients who received high ESA doses without achieving the target Hb level had worse outcomes [10–12]. The patients in those RCTs received higher ESA doses and experienced more cardiovascular comorbidities than NDCKD patients in Japan, so it was considered unreasonable to directly extrapolate those results to the management of anemia in Japanese NDCKD patients. In Japan, an observational study of dialysis patients [8] and a small interventional study in NDCKD patients [13] found that outcomes in patients with Hb levels controlled to ≥ 12 g/dL were not inferior to those in patients with Hb levels between 10 and 12 g/dL. The “Guidelines for Renal Anemia in Chronic Kidney Disease” of the Japanese Society for Dialysis Therapy (JSDT) set an Hb target of 11–13 g/dL for NDCKD patients [14], which is higher than that in guidelines published in other countries. The above RCTs used epoetin or darbepoetin alfa, but there have been no large-scale studies of the renal protective effect of improving anemia using epoetin beta pegol (continuous erythropoietin receptor activator, C.E.R.A.), which has different erythropoietin receptor-binding properties.

Based on the above considerations, a large-scale prospective investigation of the relationship between Hb level achieved during C.E.R.A. treatment and the occurrence of renal events in Japanese NDCKD patients with renal anemia was conducted to investigate the effects of anemia treatment on renal outcome in the real-world clinical setting in Japan.

## Methods

### Study design and population

This was a prospective observational study conducted in 749 institutions in Japan. Patients were enrolled from July 2012 until December 2013, and the inclusion and exclusion criteria were as follows. Inclusion criteria: patients who are (1) NDCKD patients with renal anemia (Hb < 11 g/dL), (2) receiving C.E.R.A., for the first time, regardless of history of ESA treatment, (3) not expected to transition to dialysis within 6 months from start of C.E.R.A. treatment, and (4) enrolled within 30 days after the start of C.E.R.A. treatment. Exclusion criteria: Patients who (1) have non-renal anemia, (2) had a kidney transplant in the previous year, or (3) have an estimated glomerular filtration rate (eGFR) of < 6 mL/min/1.73 m^2^.

### Anemia treatment by C.E.R.A.

C.E.R.A. (Mircera^®^ 25, 50, 75, 100, 150, 200, and 250 µg, Chugai Pharmaceutical, Tokyo, Japan) was used to treat renal anemia in this study in accordance with the dosage and administration method specified in the Japanese package insert. Target Hb levels were set at each physician’s discretion.

### Measurements

Data collected in this study: patient baseline characteristics (age, sex, CKD etiology, medical history, comorbidities); laboratory test data, and measurement dates; details of administration of C.E.R.A. and concomitant medication (Table 1); renal replacement therapy (RRT) date; adverse events.

**Table 1:** Patient baseline characteristics

Variable	*N* (%)				
	Safety analysis set (*N* = 4601)	Landmark analysis set (*N* = 2851)	Week 12 Hb (g/dL)		
			< 11 (*N* = 1793)	≥ 11 (*N* = 1058)	*P* value
Sex					
Male	2593 (56.35)	1617 (56.71)	998 (55.66)	619 (58.50)	0.1384
Age (years)					
*N*	4601	2851	1793	1058	0.9586
Mean ± SD	73.07 ± 12.59	72.71 ± 12.53	72.70 ± 12.78	72.72 ± 12.11	
BMI (kg/m^2^)					
*N*	2565	1636	1009	627	0.0184
Mean ± SD	23.05 ± 4.54	23.07 ± 4.82	23.29 ± 5.40	22.71 ± 3.69	
History of smoking	1060 (23.03)	669 (23.46)	403 (22.47)	266 (25.14)	0.0726
CKD etiology					
Chronic glomerulonephritis	903 (19.62)	567 (19.88)	371 (20.69)	196 (18.52)	0.2152
Diabetic nephropathy	1595 (34.66)	1018 (35.70)	649 (36.19)	369 (34.87)	
Nephrosclerosis	1417 (30.79)	837 (29.35)	504 (28.10)	333 (31.47)	
Other	686 (14.90)	429 (15.04)	269 (15.00)	160 (15.12)	
History of ESA therapy (last before C.E.R.A.)	1314 (28.55)	844 (29.60)	547 (30.50)	297 (28.07)	0.1687
rHuEPO	331 (7.19)	218 (7.64)	116 (6.46)	102 (9.64)	
Darbepoetin α	982 (21.34)	625 (21.92)	430 (23.98)	195 (18.43)	
Unknown	1 (0.02)	1 (0.03)	1 (0.05)	-	
Comorbidities	4410 (95.84)	2750 (96.45)	1730 (96.48)	1020 (96.40)	0.9133
Heart disease	1422 (30.90)	861 (30.19)	572 (31.90)	289 (27.31)	
Heart failure	646 (14.04)	373 (13.08)	253 (14.11)	120 (11.34)	
Angina pectoris	539 (11.71)	318 (11.15)	212 (11.82)	106 (10.01)	
Myocardial infarction	81 (1.76)	51 (1.78)	36 (2.00)	15 (1.41)	
Arrhythmia	369 (8.01)	237 (8.31)	161 (8.97)	76 (7.18)	
Other	313 (6.80)	202 (7.08)	131 (7.30)	71 (6.71)	
Brain disease	353 (7.67)	229 (8.03)	149 (8.31)	80 (7.56)	
Ischemic brain disease	310 (6.73)	202 (7.08)	132 (7.36)	70 (6.61)	
Other	50 (1.08)	32 (1.12)	21 (1.17)	11 (1.03)	
Hypertension	3789 (82.35)	2394 (83.97)	1506 (83.99)	888 (83.93)	
Malignant tumor	188 (4.08)	123 (4.31)	88 (4.90)	35 (3.30)	
Diabetes	2051 (44.57)	1319 (46.26)	828 (46.17)	491 (46.40)	
Hyperlipidemia	1929 (41.92)	1270 (44.54)	782 (43.61)	488 (46.12)	
Concomitant medication	4072 (88.50)	2565 (89.96)	1604 (89.45)	961 (90.83)	0.2385
Iron formulation	555 (12.06)	333 (11.68)	192 (10.70)	141 (13.32)	
Antihypertensive	3654 (79.41)	2309 (80.98)	1453 (81.03)	856 (80.90)	
ACEI	347 (7.54)	227 (7.96)	141 (7.86)	86 (8.12)	
ARB	2114 (45.94)	1346 (47.21)	847 (47.23)	499 (47.16)	
Calcium channel blocker	2528 (54.94)	1608 (56.40)	1015 (56.60)	593 (56.04)	
Diuretic	1671 (36.31)	1077 (37.77)	691 (38.53)	386 (36.48)	
Alpha blocker	521 (11.32)	337 (11.82)	226 (12.60)	111 (10.49)	
Beta blocker	262 (5.69)	163 (5.71)	98 (5.46)	65 (6.14)	
Other	1053 (22.88)	687 (24.09)	440 (24.53)	247 (23.34)	
Active vitamin D_3_ analog	403 (8.75)	244 (8.55)	136 (7.58)	108 (10.20)	
Hypolipidemic drug	1476 (32.07)	982 (34.44)	611 (34.07)	371 (35.06)	
Antidiabetic drug	1250 (27.16)	828 (29.04)	512 (28.55)	316 (29.86)	
Systolic blood pressure (mmHg)					
*N*	4055	2541	1599	942	0.0041
Mean ± SD	134.94 ± 20.45	134.60 ± 20.14	135.48 ± 20.62	133.11 ± 19.21	
Diastolic blood pressure (mmHg)					
*N*	4028	2524	1589	935	0.8740
Mean ± SD	70.09 ± 12.82	70.16 ± 12.73	70.12 ± 12.97	70.21 ± 12.31	
Hb (g/dL)					
*N*	4601	2851	1793	1058	< 0.0001
Mean ± SD	9.45 ± 0.92	9.48 ± 0.90	9.27 ± 0.90	9.83 ± 0.77	
Serum creatinine (mg/dL)					
*N*	4601	2851	1793	1058	0.0001
Mean ± SD	2.87 ± 1.38	2.85 ± 1.30	2.92 ± 1.32	2.73 ± 1.25	
eGFR (mL/min/1.73 m^2^)					
*N*	4601	2851	1793	1058	0.0004
Mean ± SD	20.47 ± 11.67	20.25 ± 11.08	19.69 ± 10.94	21.21 ± 11.25	
Total protein (g/dL)					
*N*	3763	2346	1472	874	0.3386
Mean ± SD	6.64 ± 0.72	6.65 ± 0.74	6.64 ± 0.74	6.67 ± 0.74	
Albumin (g/dL)					
*N*	3989	2499	1563	936	< 0.0001
Mean ± 2SD	3.62 ± 0.55	3.64 ± 0.54	3.60 ± 0.55	3.70 ± 0.53	
Ferritin (ng/mL)					
*N*	1853	1178	732	446	0.0089
Mean ± SD	166.94 ± 213.14	172.54 ± 240.75	158.24 ± 206.56	196.00 ± 286.92	
TSAT (%)					
*N*	1667	1062	661	401	0.5173
Mean ± SD	28.24 ± 12.96	28.45 ± 12.89	28.25 ± 13.07	28.78 ± 12.60	
Calcium (mg/dL)					
*N*	3562	2263	1443	820	0.0002
Mean ± SD	8.80 ± 1.22	8.83 ± 1.24	8.76 ± 1.17	8.96 ± 1.34	
Phosphate (mg/dL)					
*N*	3158	2016	1276	740	< 0.0001
Mean ± SD	3.94 ± 1.05	3.93 ± 1.13	4.01 ± 1.30	3.80 ± 0.74	
Total cholesterol (mg/dL)					
*N*	2770	1728	1093	635	0.0146
Mean ± SD	171.88 ± 42.05	171.93 ± 42.02	170.04 ± 39.84	175.16 ± 45.39	
CRP (mg/dL)					
*N*	2456	1526	966	560	0.9413
Mean ± SD	0.58 ± 1.57	0.52 ± 1.39	0.52 ± 1.37	0.53 ± 1.43	
Urine protein (spot urine) (mg/dL)					
*N*	2321	1494	919	575	0.1941
Mean ± SD	183.34 ± 256.55	187.57 ± 243.26	194.04 ± 238.07	177.24 ± 251.20	
Urine protein/creatinine ratio					
*N*	2020	1297	794	503	0.0274
Mean ± SD	5.19 ± 34.08	5.28 ± 33.78	6.93 ± 42.93	2.68 ± 4.91	
C.E.R.A. dosage (μg/4 week)					
*N*	4594	2848	1792	1056	0.1528
Mean ± SD	66.82 ± 48.99	66.35 ± 47.73	65.37 ± 44.74	68.01 ± 52.39	

N, number of patiens; SD, standard deviation; BMI, body mass index; CKD, chronic kidney disease; ESA, erythropoiesis-stimulating agent; C.E.R.A., continuous erythropoietin receptor activator; rHuEPO; recombinant human erythropoietin; ACEI, angiotensin-converting enzyme inhibitor; ARB, angiotensin receptor blocker; Hb, Hemoglobin; eGFR, estimated glomerular filtration rate; TSAT, transferrin saturation; CRP,C-reactive protein

### Endpoint

The endpoint was renal survival, defined as time until any of the following occurs: progression to RRT, serum creatinine doubling, or eGFR falling below 6 mL/min/1.73 m^2^. New adverse drug reactions (ADRs) after the start of C.E.R.A. treatment were also evaluated.

The observation period was 2 years after the start of C.E.R.A. treatment but was terminated earlier if any of the following occurred: transition to RRT, death, or discontinuation of C.E.R.A. at the physician’s discretion (excluding temporary suspensions).

### Statistical analysis

Hb levels have been reported to become steady about 12 weeks after the start of C.E.R.A. treatment [15], so, in line with the protocol, landmark analysis of the effect of Week 12 Hb level on the occurrence of renal events was performed with week 12 as the landmark time (start time).

**Fig. 1:**
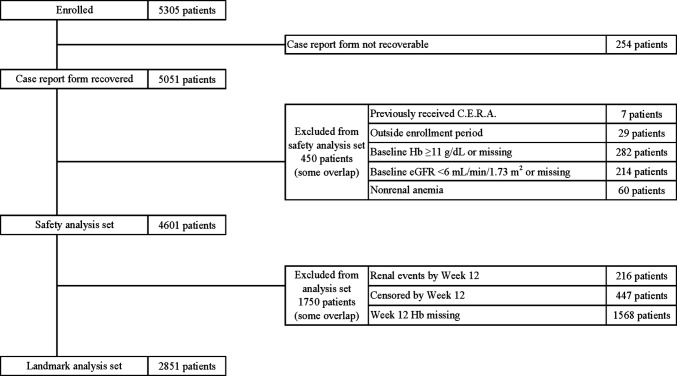
Subject disposition diagram. C.E.R.A., continuous erythropoietin receptor activator; Hb, Hemoglobin; eGFR, estimated glomerular filtration rate

The landmark analysis set was the safety analysis set minus patients who experienced renal events were censored in treatment Weeks 0 to 12, or had missing data on Hb level in treatment of week 12 (Week 12 Hb) (Fig. 1). The landmark analysis set was divided into two groups based on Week 12 Hb (< 11 g/dL, ≥ 11 g/dL), and renal survival rates were estimated by the Kaplan–Meier analysis and compared by log-rank test. In addition, univariate and multivariate Cox regression analyses were used to estimate the risk of week 12 Hb levels for renal outcomes using hazard ratio (HR) and 95% confidence interval [CI] (Table 2).

**Table 2:** Univariate and multivariate cox regression analyses with patients divided into two groups by treatment week 12 Hb level

Variable		Univariate analysis			Step-down procedure		
		Hazard ratio	95% CI	*P* value	Hazard ratio	95% CI	*P* value
Week 12 Hb (g/dL)	≥ 11	Reference			Reference		
	< 11	1.37	1.21–1.57	< 0.0001	1.26	1.05–1.51	0.0103
eGFR	5 mL/min/1.73 m^2^	0.57	0.54–0.60	< 0.0001	0.59	0.55–0.64	< 0.0001
Systolic blood pressure	10 mmHg	1.17	1.14–1.21	< 0.0001	1.11	1.06–1.15	< 0.0001
Albumin	0.5 g/dL	0.71	0.68–0.75	< 0.0001	0.7	0.65–0.75	< 0.0001
Age	10 years	0.74	0.71–0.77	< 0.0001	0.77	0.72–0.83	< 0.0001
Sex	Male	1.5	1.32–1.70	< 0.0001	1.42	1.19–1.69	< 0.0001
History of smoking	Yes	1.41	1.21–1.64	< 0.0001	–	–	–
History of ESA therapy	Yes	1.04	0.91–1.19	0.5148	–	–	–
Diet therapy	Yes	1.65	1.41–1.93	< 0.0001	1.18	0.94–1.48	0.1442
Etioligy							
Diabetic nephropathy	Yes	1.6	1.41–1.81	< 0.0001	1.25	1.00–1.55	0.0428
Nephrosclerosis	Yes	0.6	0.52–0.70	< 0.0001	–	–	–
Chronic glomerulonephritis	Yes	1.12	0.97–1.30	0.1162	–	–	–
Heart disease (medical history)	Yes	0.87	0.73–1.05	0.1707	–	–	–
Comorbidity							
Hypertension	Yes	1.63	1.33–2.00	< 0.0001	–	–	–
Heart failure	Yes	0.89	0.73–1.08	0.2561	–	–	–
Angina pectoris or myocardial infarction	Yes	0.93	0.77–1.13	0.4964	–	–	–
Diabetes	Yes	1.39	1.23–1.57	< 0.0001	–	–	–
Hyperlipidemia	Yes	1.07	0.95–1.21	0.2270	–	–	–
Concomitant medication							
Iron formulation	Yes	0.86	0.71–1.05	0.1484	0.77	0.60–1.00	0.0538
ACEIs or ARBs	Yes	1.29	1.13–1.45	< 0.0001	0.87	0.72–1.04	0.1344
Calcium channel blocker	Yes	1.9	1.66–2.17	< 0.0001	1.3	1.07–1.59	0.0069
Diuretic	Yes	1.24	1.10–1.41	0.0004	–	–	–
Activated carbon product	Yes	1.67	1.44–1.92	< 0.0001	1.27	1.04–1.54	0.0156
Active vitamin D3 analog	Yes	1.28	1.05–1.57	0.0141	1.22	0.95–1.58	0.1160
Antidiabetic drug	Yes	1.25	1.09–1.42	0.0007	0.84	0.67–1.04	0.1216
Hypolipidemic drug	Yes	1.13	0.99–1.28	0.0558	–	–	–
Sodium bicarbonate	Yes	2	1.69–2.37	< 0.0001	1.46	1.17–1.82	0.0007
Treatment Week 12 C.E.R.A. dosage	25 μg/4 week	1.08	1.05–1.11	< 0.0001	1.06	1.02–1.10	0.0025

Among variables for which < 20% of the data were missing, the above clinically significant risk factors that could conceivably be confounding factors were investigated as covariates. Multivariate Cox regression analysis was performed using a model in which covariates were selected by the step-down procedure (excluding covariates for which *P* was ≥ 0.2)

CI, confidence interval; Hb, Hemoglobin; eGFR, estimated glomerular filtration rate; ESA, erythropoiesis-stimulating agent; ACEI, angiotensin-converting enzyme inhibitor; ARB, angiotensin receptor blocker; C.E.R.A., continuous erythropoietin receptor activator

All analyses were performed using SAS version 9.4 (SAS Institute, Cary, NC, USA), and values of *P* < 0.05 were considered significant.

## Results

### Patients and baseline characteristics

Of 5305 patients enrolled, 254 for whom case report forms were not retrieved and 450 who did not meet inclusion criteria or met exclusion criteria were excluded, leaving 4601 in the safety analysis set. After excluding 1750 patients who had renal events or were censored by week 12 or who had missing data on week 12 Hb, the landmark analysis set of 2851 patients was established (Fig. 1). In these patients, the median observation period was 66.57 weeks. The landmark analysis set was divided into two groups based on week 12 Hb: the < 11 g/dL group had 1793 patients and the ≥ 11 g/dL group had 1058 patients. The median observation period was 80.14 weeks in the group with Hb level ≥ 11 g/dL and 60.00 weeks in the group with Hb level < 11 g/dL. The final observation of cumulative renal survival rate was at 158.14 weeks in the group with Hb level ≥ 11 g/dL and 164.14 weeks in the group with Hb level < 11 g/dL.

The patient baseline characteristics are shown in Table 1. Of the whole landmark analysis set, 56.7% were male, the mean age was 72.7 ± 12.5 years (there were many elderly patients, reflecting the clinical reality), 35.7% of the patients had diabetic nephropathy as the CKD etiology, and 29.6% had a history of ESA therapy before receiving C.E.R.A. There were no significant differences in sex, age, CKD etiology, medical history, comorbidity, or antihypertensive agents between the two groups.

The mean week 0 and week 12 Hb levels were respectively 9.27 ± 0.90 and 9.77 ± 0.90 g/dL in the < 11 g/dL group and 9.83 ± 0.77 and 11.79 ± 0.70 g/dL in the ≥ 11 g/dL group. The mean week 0 and week 12 eGFR values were respectively 19.69 ± 10.94 and 18.98 ± 12.00 mL/min/1.73 m^2^ in the < 11 g/dL group and 21.21 ± 11.25 and 21.02 ± 12.01 mL/min/1.73 m^2^ in the ≥ 11 g/dL group.

### Hb level achieved and C.E.R.A. dosage

In the ≥ 11 g/dL group, Hb level remained around 11 g/dL at all time points after week 12. In the < 11 g/dL group, Hb level stayed around 10 g/dL after week 16.

**Fig. 2:**
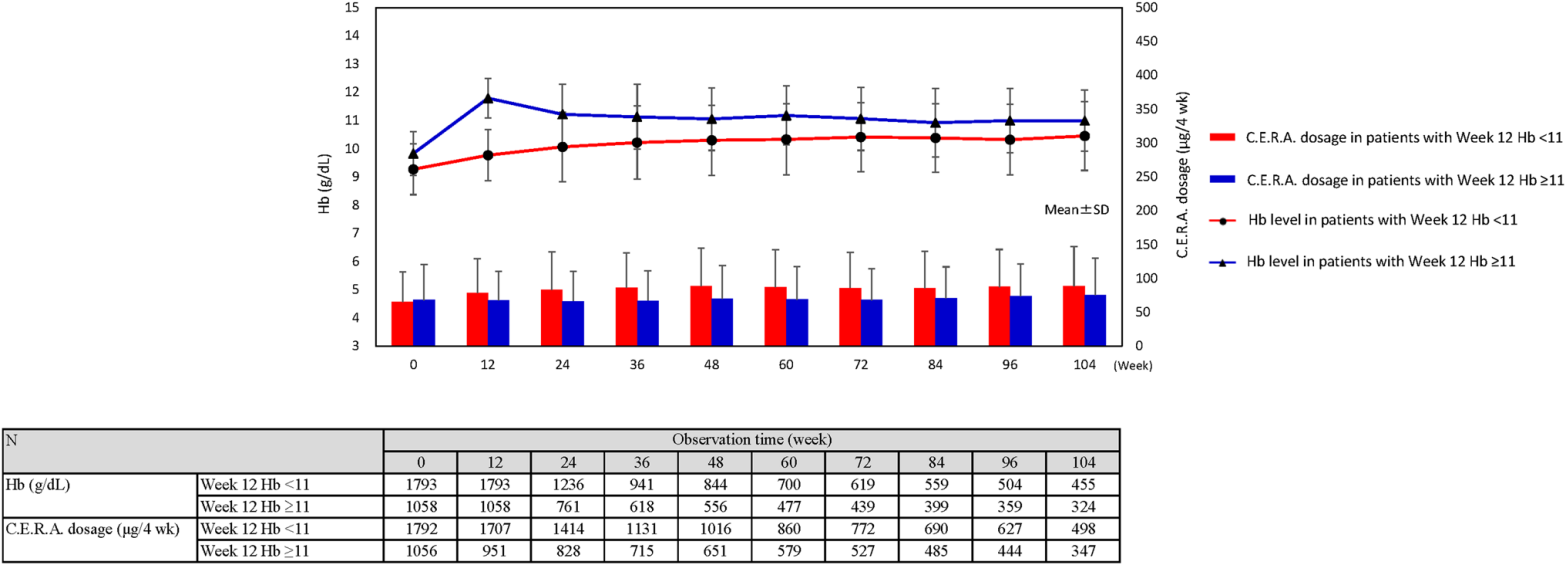
Time courses of C.E.R.A. dosage and Hb level with patients divided into two groups by treatment week 12 Hb level. C.E.R.A., continuous erythropoietin receptor activator; Hb, Hemoglobin; N, number of patients; SD, standard deviation

The week 12 C.E.R.A dosage in the ≥ 11 g/dL group (67.80 ± 42.47 µg/4 week) was significantly lower than in the < 11 g/dL group (78.55 ± 50.43 µg/4 week) (*P* < 0.0001). The range of mean dosages during the observation period was 64.88–75.50 µg/4 week in the ≥ 11 g/dL group and 78.55–88.89 µg/4 week in the < 11 g/dL group, remaining roughly the same as in week 12 (Fig. 2).

### Endpoint

According to landmark analysis starting from treatment week 12, renal events occurred in 693 patients (38.65%) in the < 11 g/dL group and 338 patients (31.94%) in the ≥ 11 g/dL group.

**Fig. 3:**
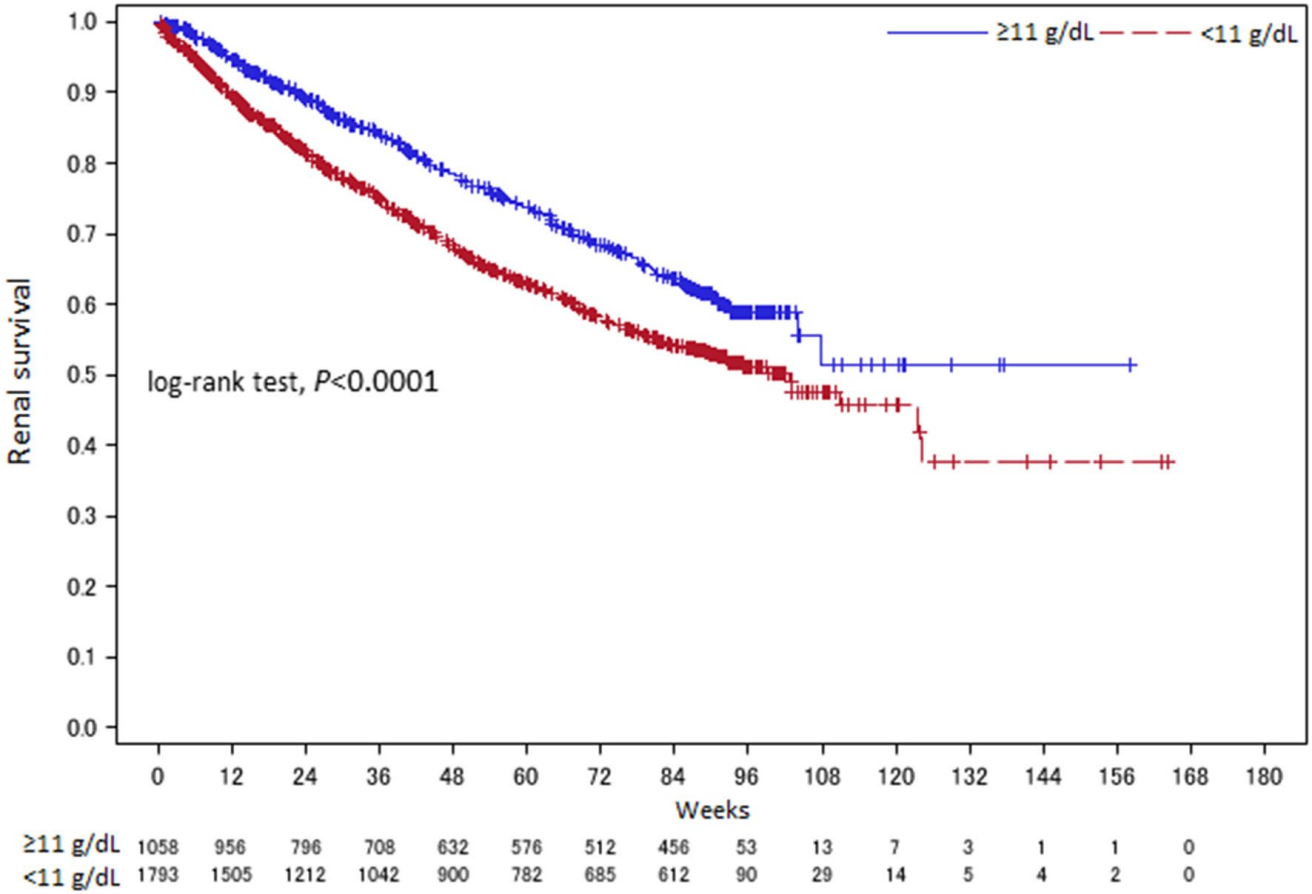
Kaplan–Meier analysis with patients divided into two groups by treatment Week 12 Hb level. Week 0: C.E.R.A. treatment week 12 was used as the landmark time (start time)

The overall 2 year renal survival rate by Kaplan–Meier analysis was 37.57% in the < 11 g/dL group and 51.47% in the ≥ 11 g/dL group, so it was significantly higher in the ≥ 11 g/dL group (*P* < 0.0001, log-rank test) (Fig. 3).

Investigation of the HR for renal events using multivariate Cox regression analysis also revealed a significantly higher risk of renal events in the < 11 g/dL group compared to the ≥ 11 g/dL group (HR 1.26; 95% CI 1.05–1.51; *P* = 0.0103). High eGFR, high albumin, and advanced age significantly decreased the risk of renal events, whereas high systolic blood pressure, male sex, diabetic nephropathy, calcium channel blockers, activated carbon product, sodium bicarbonate, and higher C.E.R.A. dose in week 12 significantly increased the HR for renal events (Table 2).

**Fig. 4:**
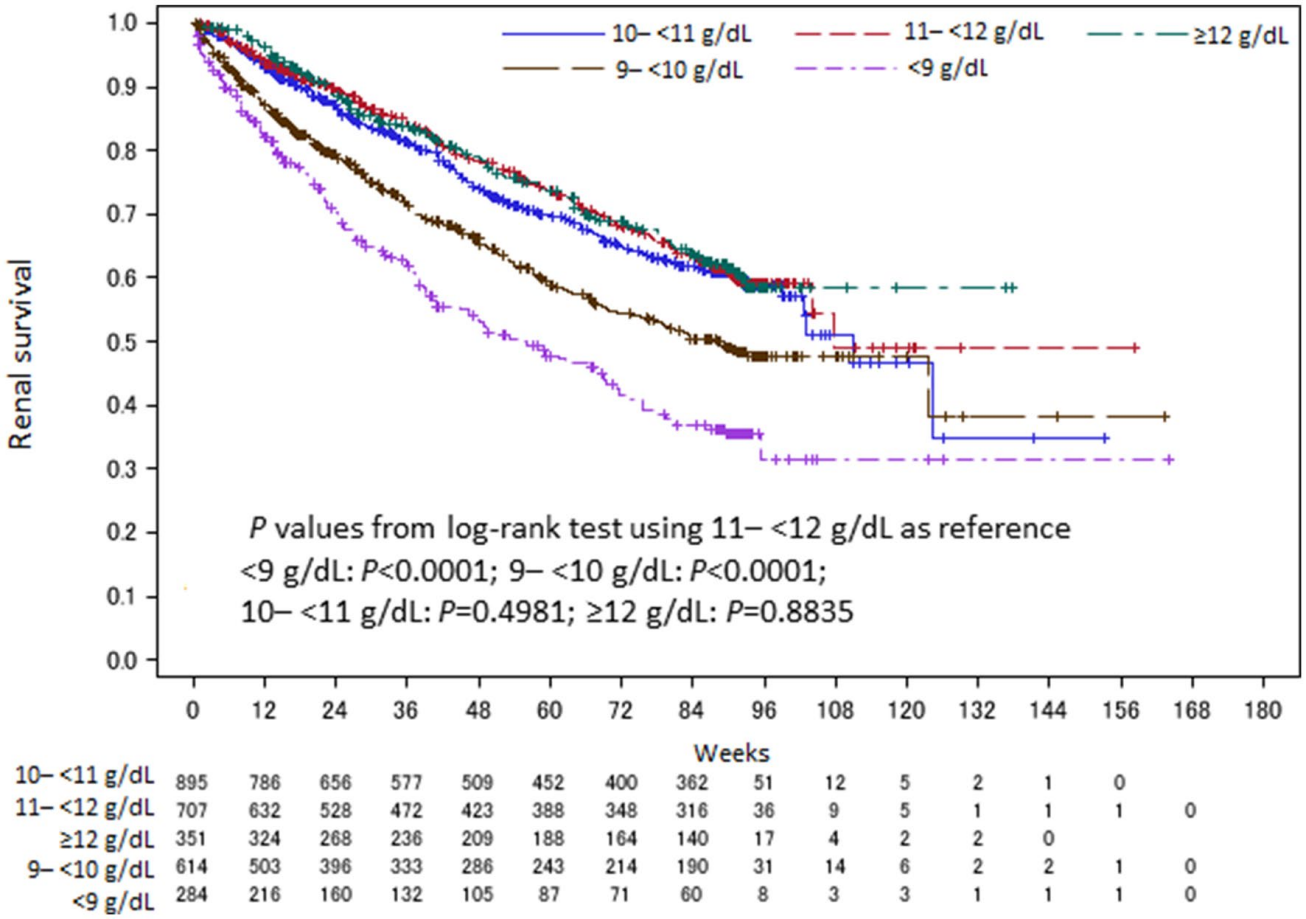
Kaplan–Meier analysis with patients divided into five groups by treatment Week 12 Hb level. The overall renal survival rate by Kaplan–Meier analysis was 31.43% in the < 9 g/dL group, 38.08% in the 9 to < 10 g/dL group, 34.98% in the 10 to < 11 g/dL group, 49.00% in the 11 to < 12 g/dL group, and 58.60% in the ≥ 12 g/dL group. Week 0: C.E.R.A. treatment Week 12 was used as the landmark time (start time). Hb, Hemoglobin; N, number of patients

Kaplan–Meier analysis of the patients divided into five groups based on Week 12 Hb (< 9, 9 to < 10, 10 to < 11, 11 to < 12, and ≥ 12 g/dL) showed that renal outcomes were poorer with Hb levels < 10 g/dL (Fig. 4), and multivariate Cox regression analysis (step-down procedure) revealed a significantly higher risk of renal events in the < 9 g/dL group (HR 1.89; 95% CI 1.39–2.56; *P* < 0.0001) and the 9–<10 g/dL group (HR 1.52; 95% CI 1.19–1.94; *P* = 0.0006) compared to the 11–<12 g/dL (reference) group (data not shown).

To investigate the effect of the Hb level not only at a single point (Week 12) but also during its subsequent course, the relationship between length of time with Hb ≥ 11 g/dL after start of C.E.R.A. treatment and renal events was investigated using multivariate Cox regression analysis. Dividing the patients into three groups based on time with Hb ≥ 11 g/dL during the first year of C.E.R.A. treatment (< 3 month, 3 to < 6 month, and 6–12 month) and analyzing the risk of renal events occurring after the first year of treatment revealed a significantly lower risk of renal events in the 6–12 month group (HR 0.72; 95% CI 0.55–0.93; *P* = 0.0135) compared to the < 3 month group (data not shown).

### Subgroup analyses

**Fig. 5:**
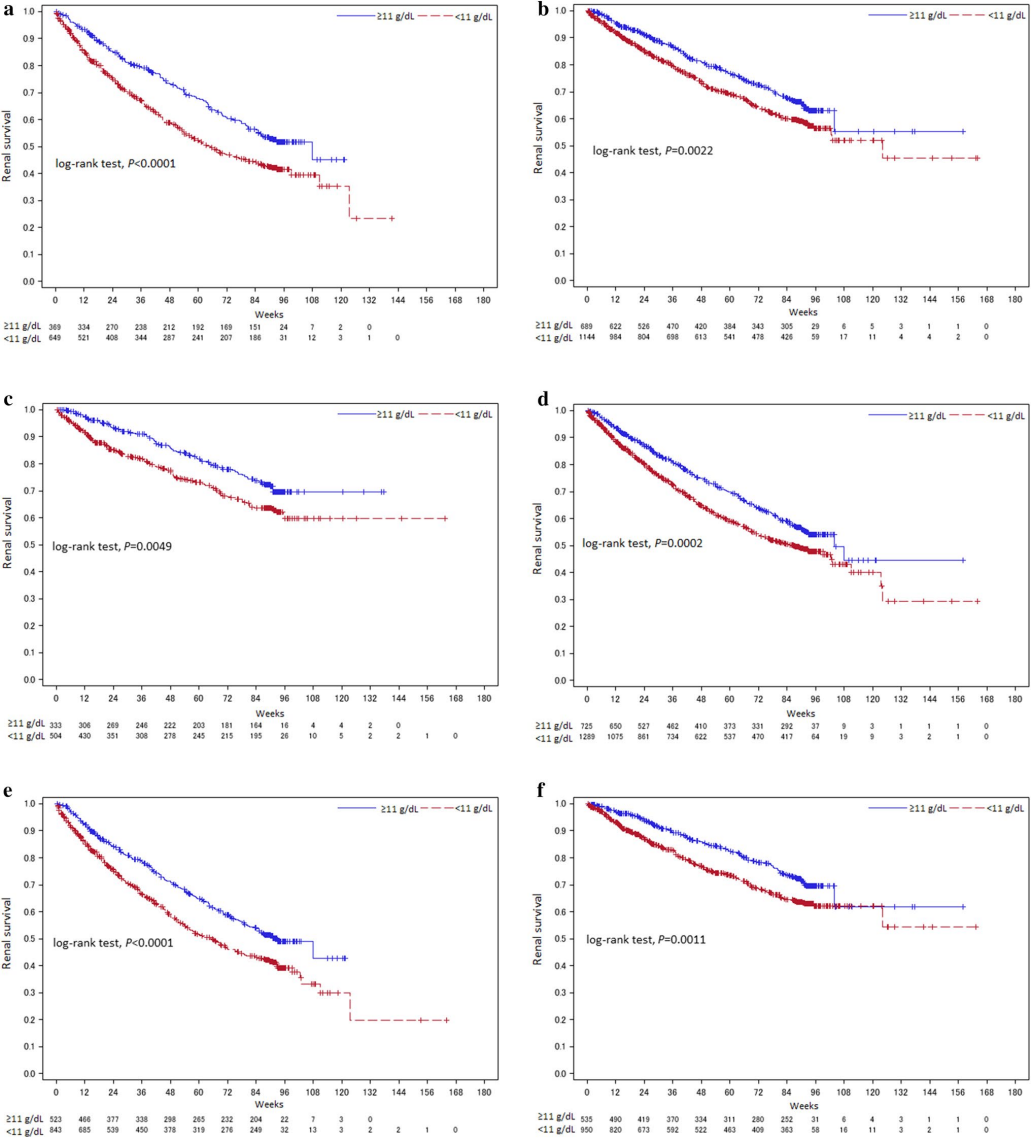
Kaplan–Meier analysis (by CKD etiology and age) with patients divided into two groups by treatment Week 12 Hb level. **a** Patients with diabetic nephropathy as the CKD etiology. **b** Patients without diabetic nephropathy as the CKD etiology. **c** Patients with nephrosclerosis as the CKD etiology. **d** Patients without nephrosclerosis as the CKD etiology. **e** Patients aged < 75 years. **f** Patients aged ≥ 75 years. **a** Overall renal survival rate by Kaplan–Meier analysis was 23.45% in the < 11 g/dL group and 45.11% in the ≥ 11 g/dL group for patients with diabetic nephropathy as the CKD etiology. **b** Overall renal survival rate by Kaplan–Meier analysis was 45.33% in the < 11 g/dL group and 55.13% in the ≥ 11 g/dL group for patients without diabetic nephropathy as the CKD etiology. **c** Overall renal survival rate by Kaplan–Meier analysis was 59.69% in the < 11 g/dL group and 69.58% in the ≥ 11 g/dL group for patients with nephrosclerosis as the CKD etiology. **d** Overall renal survival rate by Kaplan–Meier analysis was 29.21% in the < 11 g/dL group and 44.57% in the ≥ 11 g/dL group for patients without nephrosclerosis as the CKD etiology. **e** Overall renal survival rate by Kaplan–Meier analysis was 19.85% in the < 11 g/dL group and 42.81% in the ≥ 11 g/dL group for patients aged < 75 years. **f** Overall renal survival rate by Kaplan–Meier analysis was 54.25% in the < 11 g/dL group and 61.70% in the ≥ 11 g/dL group for patients aged ≥ 75 years. Week 0: C.E.R.A. treatment week 12 was used as the landmark time (start time). Hb, Hemoglobin; N, number of patients

The effect of Week 12 Hb on the occurrence of renal events was also investigated in CKD etiology and age subgroups. Kaplan–Meier analysis showed that patients in the ≥ 11 g/dL group had significantly lower risk of renal events compared to the < 11 g/dL group across the subgroups with or without diabetic nephropathy, with or without nephrosclerosis, and with age ≥ 75 or < 75 years (see Fig. 5 for *P* values).

**Fig. 6:**
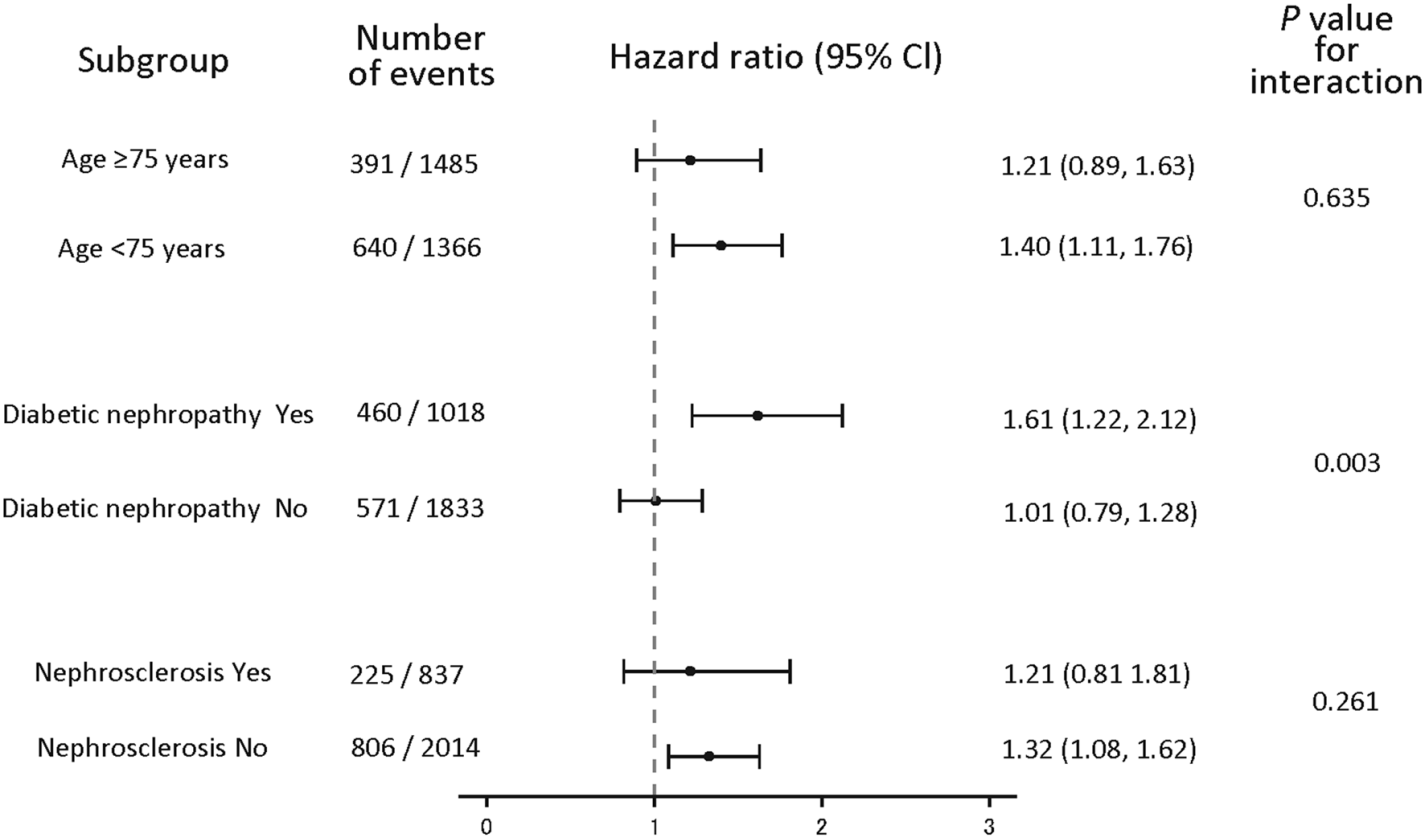
Subgroup analyses by age and CKD etiology. Hazard ratios and 95% CI were adjusted for multivariate Cox regression analysis. Hazard ratio of group with Week 12 Hb level < 11 g/dL when hazard ratio of group with Week 12 Hb level ≥ 11 g/dL is 1. CI, confidence interval

Multivariate Cox regression analyses also showed that favorable effects of higher Week 12 Hb level on renal outcomes were observed across patient subgroups with age ≥ 75 or < 75 years (*P* = 0.635 for interaction) and with or without nephrosclerosis (*P* = 0.261 for interaction). However, a similar effect was not observed in the patient subgroups with or without diabetic nephropathy (*P* = 0.003 for interaction) (Fig. 6).

### Safety analyses

ADRs occurred in 195 (4.23%) of the 4601 patients in the safety analysis set. Among the 2851 patients in the landmark analysis set, ADRs occurred in 77 patients (4.29%) in the < 11 g/dL group and 45 patients (4.25%) in the ≥ 11 g/dL group; Hb level did not affect the incidence of ADRs. Serious ADRs occurred in 39 patients (2.17%) in the < 11 g/dL group and 22 patients (2.07%) in the ≥ 11 g/dL group (Table 3).

**Table 3:** Adverse drug reactions

Category	Safety analysis set (*N* = 4601)		Landmark analysis set (*N* = 2851)		Week 12 Hb			
					< 11 (*N* = 1793)		≥ 11 (*N* = 1058)	
	Serious	Total	Serious	Total	Serious	Total	Serious	Total
No. of patients with ADRs	98	195	61	122	39	77	22	45
No. of occurrences of ADRs	124	238	77	147	49	92	28	55
ADR incidence (%)	2.12	4.23	2.13	4.27	2.17	4.29	2.07	4.25

No., number of patiens; ADR, adverse drug reaction; Hb, Hemoglobin

## Discussion

The relationship between treating renal anemia with ESAs and renal outcomes in NDCKD patients has been investigated in various observational studies and secondary analyses of large-scale RCTs over the past 2 decades. The evidence level is high, particularly from large-scale RCTs (e.g., the CHOIR, CREATE, and TREAT), but this evidence cannot be directly extrapolated to the Japanese clinical setting, because the high ESA doses and very high rates of cardiovascular comorbidities in these studies differ greatly from those found in Japan. Furthermore, because these studies used epoetin and darbepoetin alfa, the effect of C.E.R.A. to treat renal anemia on renal outcome remained unclear. We, therefore, conducted a large-scale prospective observational study to clarify this effect in Japanese NDCKD patients under actual-use conditions.

The results confirmed two crucial points. The first is that landmark analysis of week 12 Hb showed a significantly lower risk of renal events in the ≥ 11 g/dL group compared to the < 11 g/dL group (Fig. 3; Table 2). Furthermore, dividing the patients into five groups based on week 12 Hb revealed significantly higher risk of renal events in the < 9 and 9 to < 10 g/dL groups compared to the 11–<12 g/dL reference group (Fig. 4). We, therefore, consider favorable renal outcomes more likely if the Hb level can be kept to at least ≥ 10 g/dL and ideally ≥ 11 g/dL. Investigation of the relationship between the length of time that Hb was ≥ 11 g/dL and the onset of renal events suggested that achieving an Hb level ≥ 11 g/dL for ≥ 6 months with C.E.R.A. further decreases the risk of renal events, as shown by Luca et al. [16]. Although the 2012 KDIGO guidelines [9] decreased the upper limit of the target Hb level to 11.5 g/dL, emphasizing the results of the CHOIR, CREATE, and TREAT studies, our results support the target maintenance Hb level in NDCKD patients (11 to < 13 g/dL) stated in the 2015 JSDT “Guidelines for Renal Anemia in Chronic Kidney Disease” [14] for patient populations (such as CKD patients in Japan) that receive lower ESA doses and have fewer cardiovascular comorbidities than CKD patients in the United States.

Furthermore, in multivariate Cox regression analysis of subgroups with or without diabetic nephropathy, with or without nephrosclerosis, and with age ≥ 75 or < 75 years, interaction was observed in the patient subgroups with or without diabetic nephropathy (*P* = 0.003 for interaction) (Fig. 6). The status of atherosclerotic lesions in multiple organs including the kidneys may differ in the patient subgroups with or without diabetic nephropathy; it might have affected this interaction. In addition, it seems to be relevant that higher Hb level would cancel the benefit from ESA treatment as previously shown in the CHOIR and TREAT studies. This could warrant further investigation of the usefulness of achieving an Hb level ≥ 11 g/dL to delay renal outcomes in these patient groups.

The second point is that at Week 12 C.E.R.A dosage in the ≥ 11 g/dL group (67.80 ± 42.47 µg/4 week) was significantly lower than that in the < 11 g/dL group (78.55 ± 50.43 µg/4 week) (*P* < 0.0001). And, thereafter, they remained roughly the same as in week 12 (Fig. 2). Recently, Tsuruya et al. reported the results of a pooled analysis of C.E.R.A. clinical studies in Japan. They divided patients into two groups based on median erythropoietin resistance index (ERI) in Week 12 of C.E.R.A. treatment, as a measure of ESA responsiveness, and found that renal outcome (initiation of dialysis or 30% decrease in eGFR) was significantly poorer in the poor-response group compared to the good-response group [17]. Our study did not investigate ERI, but the C.E.R.A. dose in the ≥ 11 g/dL group tended to be lower than in the < 11 g/dL group throughout the observation period. This suggests a possible relationship between C.E.R.A. responsiveness and renal outcome. We are aware that ESA hyporesponsiveness is an unstudied issue of ESA therapy for renal anemia and look forward to the results of a prospective comparative study of the relationship between ERI and improvement of renal outcome.

In terms of safety evaluation, there were no major differences in the incidence of ADRs related to elevated Hb (e.g., myocardial infarction, angina pectoris, cerebral infarction, cerebral hemorrhage, aortic dissection, and hypertension). Therefore, achieving an Hb level ≥ 11 g/dL is not considered to present any safety issues in actual clinical practice.

These results should be interpreted with the study’s limitations in mind. First, this was an observational study, so setting the target Hb level was the responsibility of the physicians, not the result of an intervention. Furthermore, we cannot exclude the possibility of reverse causality regarding several covariates (calcium channel blockers, activated carbon product, and sodium bicarbonate) used in the multivariate Cox regression analysis. Second, although the patient baseline characteristics, Hb levels, and other data, shown in Table 2, were adjusted for using multivariate Cox regression analysis, adjustment for urine protein, urine creatinine, and C-reactive protein was not performed, because ≥ 20% of the data were missing. If factors with ≥ 20% of data missing are included in analysis as covariates, it can introduce bias in multivariate Cox regression analysis results obtained for the remaining patients. Furthermore, the assessment of responsiveness to ESAs in terms of ERI was not performed. Finally, the use of landmark analysis starting at treatment Week 12 meant that 1750 patients in the safety analysis set were excluded. Data on week 12 Hb levels were missing for 1568 of these patients. Once monthly measurement of Hb levels was specified in advance in the protocol, but, because this was an observational study under actual-use conditions, monthly patient hospital visits, and laboratory testing could not be enforced. Nevertheless, there is thought to be no selection bias, because (1) there were no noteworthy clinical differences in patient baseline characteristics between the 4601 safety analysis set patients and the 2851 landmark analysis set patients and (2) the incidence of renal events in the landmark analysis set was roughly the same as previously reported incidences of renal events [13, 18].

The strengths of the study are as follows. First, the results are directly applicable into real-world clinical practice, because a large number of patients and institutions participated, reflecting the clinical situation in Japan. Second, despite being an observational study, adequate patient information and test data were obtained, and reliable analysis was performed. Finally, the study complied with the Japanese Ministry of Health, Labour and Welfare’s GPSP standards, and there was thorough adverse event surveillance. In the real-world clinical setting, we consider it desirable to set Hb treatment targets based on the patient’s condition, with reference to the results of this study and based on recommendations in the 2015 JSDT “Guidelines for Renal Anemia in Chronic Kidney Disease” [14].

## Conclusion

This study investigated the relationship between the achieved Hb level and renal outcome when renal anemia is treated with C.E.R.A. in Japanese NDCKD patients in the actual clinical setting.

The results suggest that renal outcomes are better when the achieved Hb level is ≥ 11 g/dL than when it is < 11 g/dL, but an RCT is needed to verify these results.

## Electronic supplementary material

Below is the link to the electronic supplementary material.

Supplementary material 1 (DOCX 34 KB)
