# Supplementary material for: Effect of achieved hemoglobin level on renal outcome in non-dialysis chronic kidney disease (CKD) patients receiving epoetin beta pegol: MIRcerA CLinical Evidence on Renal Survival in CKD patients with renal anemia (MIRACLE-CKD Study)
Source: Clin Exp Nephrol. 2018 Oct 5;23(3):349–61. doi: 10.1007/s10157-018-1649-0 (PMC6394571; doi:10.1007/s10157-018-1649-0)
Supplement: Supplementary file 1 — Supplementary material 1 (DOCX 34 KB) [file 10157_2018_1649_MOESM1_ESM.docx]

# **Appendix**

The MIRACLE-CKD study group comprised the following 749 institutions in Japan:

Hokkaido: Jinzonaika Megumi Clinic; Hokuou Hospital; Shinsapporo Hospital of Cardiology; Souen Central Hospital; Sapporo Medical University Hospital; Sapporo Hokuyu Hospital; Shinsapporo Seiryou Hospital; Nippon Telegraph and Telephone East Corporation Sapporo Hospital; Federation of National Public Service Personnel Mutual Aid Association Tonan Hospital; Miyanosawa Clinic of Internal Medicine and Cardiology; Sapporo City General Hospital; Nishikimachi medical clinic; Teinekeijinkai Hospital; Akutsu medical clinic; Shinkotoni Family Clinic; Hokkaido University Hospital; KOTONI NEPHROLOGY AND UROLOGY CLINIC; Kotoni Heart Internal Medicine･Dialysis Clinic; Nikko Memorial Hospital; Midorino Clinic; Municipal Bibai Hospital; Iwamizawa Municipal General Hospital; Oji General Hospital; Tsubetsu Hospital; Kitami Cardiorenal Clinic; Nemuro Kyoritsu Hospital; Koga Hospital; Abashiri-Kosei General Hospital; Goryoukaku nephroclinic; Hakodate Municipal Hospital; Takikawa Municipal Hospital; Sunagawa City Medical Center; Kitasaito Hospital; Municipal Ashibetsu Hospital. Iwate: Department of Urology Iwate Medical University; Hidakami-chuou Clinic; Honda Clinic; Iwate prefectural Isawa Hospital; Medical corporation OBARA CLINIC; Nakasato Clinic; Department of Nephrology and Rheumatology Iwate Prefectual Central Hospital; Hiroshi Tamura's Hilltop Clinic; AKASAKA HOSPITAL. Akita: ogachi central hospital urology; Akita Rosai Hospital; Oga Minato Municipal Hospital; Akita Kousei Medical Center; Nakadori General Hospital; Akita Urologic Clinic; Seihukai Seiwa Hospital; Saga clinic; Kita Akita City Medical office. Aomori: Kanita Clinic; Hachinohe Heiwa Hospital; Sato Toriage Clinic; Medical Court Hachinohe West Hospital; TSUGARU GENERAL HOSPITAL; Kondoh Hospital; Department of Cardiology and Nephrology, Hirosaki University Graduate School of Medicine; Department of pediatrics, Hirosaki University Hospital; Department of Endocrinology and Metabolism, Hirosaki University Graduate School of Medicine. Fukushima: Tokiwa-kai Jyoban Hospital; TAKEDA general hospital (Aizu-Wakamatsu City). Miyagi: Japan Community Health Care Organization Sendai Hospital; Tohoku Kosai Miyagino Hospital; Sendai jin hinyoukika; Hotta Osamu Clinic; Department of Nephrology, Endocrinology, and Vascular Medicine, Tohoku University Hospital; Japanese Red Cross sendai Hospital; Eijinkai Hospital. Yamagata: Yamagata City Hospital SAISEIKAN. Saitama: Iwatsuki minami hospital; Saitama Red Cross Hospital; Murata Clinic; Miura Clinic; Kaneda Clinic; sashiogi hospital; JCHO Saitama Medical Center; Tokutake iine; Minami souka clinic; Sugiura Clinic; Saito Kinen Clinic; Dokkyo Medical University Koshigaya Hospital; saitama eastern cardiovascular center; Mashiko Hospital; Saitama Tukuba Hp; Kawaguchi Municipal Medical Center; Warabi Municipal Hospital; Soka Municipal Hospital; Toda Central General Hospital; Hamasaki Clinic; Fujidouri Owada naika Clinic; Uno Clinic; Saitama Tukinomorri Clinic; AGEO CENTRAL GENERAL HOSPITAL; asahi shinryojo; Saitama Medical University Hospital; Gyoda Central General Hospital; Kodama Chuuou Hospital; Saitama Jikei Hospital; Kouseikai General Clinic & Hospital; KUBOJIMA CLINIC; kobayashi naika iin; Musashidai Hospital; Ogawa Clinic; Horinouchi Hospital; KANOUZAWA CLINIC; IMAMAKI CLINIC; KAWATSURU PLAZA CLINIC; Tanaka Clinic; ASAKA KOSEI HOSPITAL; Saitama Medical Center, Saitama Medical University; Shiomi hospital. Gunma: MOTOJIMA GENERAL HOSPITAL; Seseragi Hospital; Saiseikai Maebashi Hospital; Akagi Clinic; Heisei Hidaka Clinic; Fujioka general Hospital. Tochigi: Saiseikai Utsunomiya Hospital; HAGA RED CROSS HOSPITAL; Okuda Clinic; IKENAGA JIN - CLINIC; OGATA CLINIC; Yukawa Clinic of Internal Medicine; UENO CLINIC; SUDA CLINIC; KUROSU HOSPITAL; Dokkyo Medical University. Ibaraki: Ibaraki Prefectural Central Hospital; Yamaki Medical Clinic; Juo Higashino Clinic; Naka Kinen Clinic; TAKI hospital; KURANO CLINIC; Oasis Clinic; Tokyo Medical University Ibaraki Medical Center; Ami Kobayashi Clinic; Kinu Medical Association Hospital; Itabashi Diabetes Center & Dermatology Clinic; University of Tsukuba,Department of Nephrology,Division of clinical medicine, Faculty of Medicine; Shiigai Clinic. Niigata: Niigata Prefectural Yoshida Hospital; Shinraku-en Hospital; Niigata prefectural Sakamachi Hospital; SADO GENERAL HOSPITAL; Niigata Medical Center; Ojiya General Hospital; Itoigawa General Hospital; Keinan General Hospital; Kashiwazaki General Hospital and Medical Center. Nagano: Nagano Municipal Hospital; Nagano-red cross-hospital; Shimori medical clinic; Hokushin general hospital; Kitano Hospital; Ueda Renal Clinic; Chikuma Central Hospital; Nagano Chuo Hospital; Ideura Medical Clinic; INA CENTRAL HOSPITAL; SUWA CENTRAL HOSPITAL; Shinshu University Hospital. Yamanashi: SYAKAIIRYOUHOUJIN KYOUNANNKAI KYOUNANNBYOUIN; Yamanashi Red Cross Hospital; Kodate Clinic; Sakurahayashi-Jin-Naika Clinic; University of Yamanashi. Tokyo: Department of Internal Medicine, Keio University School of Medicine; Diabetes Center Tokyo Women's Medical University School of Medicine; Japan Community Health care Organization Tokyo Yamate Medical Center; National Center for Global Health and Medicine; Kosei Hospital; Kami-itabashi Hospital; Tokyo Metropolitan Ohtsuka Hospital; Tokyo Metropolitan Police Hospital; Tokyo-Kita Medical Center; Department of Internal Medicine Teikyo University School of Medicine; Depatment of Nephrology Toho University Ohashi Medical Center; Ikegami General Hospital; Division of Nephrology, Department of Medicine, Showa University School of Medicine; Department of Nephrology, Toho University Faculty of Medicine, Tokyo, Japan; Department of Nephrology,Tokyo Metropolitan Hiroo Hospital; National Hospital Organization Tokyo Medical Center; Nihon University Hospital; Tokyo Medical and Dental University; Mitsui Memorial Hospital; The University of Tokyo School of Medicine; Department of Nephrology, Juntendo University Faculty of Medicine; Tokyo Saiseikai Central Hospital; Juntendo Tokyo Koto Geriatric Medical Center; St. Luke's International Hospital; Tokyo Metropolitan Bokutoh Hospital; Division of Nephrology and Hypertension, Department of Internal Medicine, The Jikei University School of Medicine; Kidney Clinic Setagaya; Koshikawa Clinic; TOMONARI CLINIC; MURABAYASHI CLINIC; Yoyogiyamashita Clinic; Fukasawa 1 Choume Clinic; Inoue Naikaiinn; Sangenjaya Hospital; Kaburaki Clinic; Akimoto yoshi medical clinic; Kisen Hospital; ITSUKIKAI Heart Clinic; Nishi Clinic (Taito-ku); Keijin hospital; Kobayashi Internal Medicine Clinic; Kambara clinic; OKM Okamoto Medical Clinic; Jimba clinic; Ekimae Tsunoda; Takeshita clinic; Noto clinic; Kugayama Clinic; Ogigubo Naika Clinic; Kogure clinic; Nakano Chuo clinic; Seki clinic; Keiai hospital; Saitou Clinic; Morita Medical Office; Oizumi Central Clinic; Keiai fuzoku clinic; Tsukushinbo clinic; Kusunoki Clinic; Toshimashowa Hospital; Ikebukuro Kuno Clinic; KITAHONDORI INTERNISTCLINIC; Yasui Clinic; Tsuchiya Clinic; Inagi Kidney Clinic; Hino Municipal Hospital; Tama-center Mirai Clinic; Inagi Municipal Hospital; National Hospital Organization Disaster Medical Center; Nagakubo Hospital; Ome Municipal General Hospital; Musashino Yowakai Hospital; Kyohsaikai Sakurai Hospital; The 1st Department of Internal Medicine, Kyorin University, School of Medicine; Chofu Touzan Hospital; Musashino Red Cross Hospital; Takiyama hospital; Tsutsujigaoka jindai clinic; KAMADA CLINIC; KIYOSENOMORI COMMUNITY CLINIC. Chiba: UEDA CLINIC; MIHAMA HOSPITAL; Chiba-East National Hospital; Japan Community Health care Organization Chiba Hospital; Chiba University Hospital; Chiba Hokuso Naika Clinic; Asahi General Hospital; Nippon Medical School Chiba Hokusoh Hospital; Kodama Medical Clinic; Japanese Red Cross Narita Hospital; Hotaruno Central Naika; Yamanouchi Hospital; Kimitsu Chuo Hospital; Juntendo University Urayasu Hospital; Tokyo Bay Urayasu Ichikawa Medical Center; TOKYO DENTAL COLLEGE Ichikawa General Hospital; Tokatsu-Clinic Hospital. Kanagawa: National Hospital Organization Yokohama Medical Center; Yokohama City Minato Red Cross Hospital; Saiseikai Yokohamashi Nanbu Hospital; Yokohama Minami Clinic; Yokohama Municipal Citizen's Hospital; Showa University Northern Yokohama Hospital; Heiwa Hospital; Showa University Fujigaoka Hospital; Saiseikai Yokohamashi Tobu Hospital; General Takatsu Central Hospital; Kanto Rosai Hospital; Kawasaki Saiwai Clinic; Kawasaki Municipal Ida Hospital; KOUKAN CLINIC; Yokohama Minami Kyousai Hospital; Yokohama City University Medical Center; Yokohama Sakae Kyosai Hospital; Nukada Memorial Hospital; Yokosuka City Hospital; Yokohama City University, School of Medicine,; Shonan Kamakura General Hospital; JINKEN CLINIC; Okabayashi Clinic; Yamato Municipal Hospital; TOMEI ATSUGI HOSPITAL; KITASATO UNIVERSITY; Kino iin; Hirose Hospital; Minamiyamato Hospital; Ebina General Hospital; Brite Hill Clinic; Japanese Red Cross Sagamihara Hospital; Japan Community Health care Organization Sagamino Hospital; Atsugi City Hospital; Chigasaki Medical Clinic; Tokai University School of Medicine; Tokai University Oiso Hospital; Chigasaki Municipal Hospital; Hishiki Clinic; Numata Internal Medical Clinic; OIKAWA CLINIC; Takahashi Internal Medical Clinic. Aichi: Nagoya Medical Center; Nagoya Daini Red Cross Hospital; Nagoya City University; Nagoya City East Medical Center; Tsushima City Hospital; Chubu Rosai Hospital; Japan Community Health Care Organization Chukyo Hospital; Nagoya Central Hospital; Kawana General Hospital; Masuko Memorial Hospital; owarinishi clinic; Fujiyamadai Clinic; Tosei General Hospital; Aichi Medical University; Kasugai Municipal Hospital; Handa City Hospital; Fujita Health University; Shinseikai Daiichi Hospital; Nishichita General Hospital; Anjo Kosei Hospital; TOYOKAWA CITY HOSPITAL; Toyohashi Municipal Hospital. Gifu: Gifu Prefectural General Medical Center; Tokai Central Hospital; HAKUAIKAI Hospital; Oogaki Central Hospital; Tohno Kousei Hospital. Mie: Matsusaka municipal hospital; Mie University Hospital; Ise Red Cross Hospital; Takeuchi Hospital; Tsu-Chuo-Clinic; Kinan Hospital; Okano Clinic; Okanami General Hospital; Tomidahama Hospital; Suzuka Kidney Clinic; IGA City General Hospital; Kuwana East Medical Center. Shizuoka: Yaizu City Hospital; Fujieda Municipal General Hospital; Shizuoka Saiseikai General Hospital; KITAGAWA Medical Clinic; Shizuoka City Shizuoka Hospital; Hamamatsu University School of Medicine; Iwata City Hospital; Hamamatsu Medical Center; Enshu Hospital; Nozomi Memorial Shimoda Cardiovascular Nephro Clinic; Juntendo University Shizuoka Hospital. Ishikawa: Hakui Hospital; Noto General Hospital; Division of Nephrology, Kanazawa University Hospital; Division of Rheumatology, Kanazawa University Hospital; Public Central Hospital of Matto Ishikawa; Kanazawa Municipal Hospital; Kaga Medical Center; Kanazawa Arimatsu Hospital; Yonejimaiin. Fukui: Fukui-ken Saiseikai Hospital; Fujita Memorial Hospital; Hosokawa medical clinic; Yoshimura Medical Clinic; ARITSUKA IIN. Toyama: Toyama Rosai Hospital; Naito Medical Clinic; Takaokaminami Hospital; Toyama University Hospital; Tonami General Hospital; FUJIKOSHI HOSPITAL. Osaka: Toyonaka Municipal Hospital; National Cerebral and Cardiovascular Center; Ikeda City Hospital; Osaka University Hospital; Department of Nephrology,Osaka Medical College Hospital; Sanko Hospital; Department of Urology,Osaka Medical College Hospital; Nishi Clinic (Osaka City); Osaka National Hospital; Osaka City General Hospital; Osaka Red Cross Hospital; Osaka General Medical Center; Taniguchi Clinic; Japan Community Health care Organization Osaka Hospital; Chibune General Hospital; KITANO HOSPITAL, TAZUKE KOFUKAI MEDICAL RESEARCH INSTITUTE; Sumitomo Hospital; Kawachi medical clinic; Fujii Hospital; Rinku General Medical Center; Shiroyama Hospital; Fujidera Keijinkai Clinic; Seikeikai Hospital; National Hospital Organization Osaka Minami Medical Center; Kindai University Hospital; Iwamoto clinic; Kindai University Sakai Hospital; Tanakakitanoda Hospital; Kato Medical Clinic Minato; Seichokai Fuchu hospital; Department of Medicine Ⅱ Kansai Medical University; Matsushita Memorial Hospital; Higashikouri Hospital; Ibuki Clinic; shiraiwa medical clinic. Hyogo: Kobe Asahi Hospital; Hara Genitourinary Hospital; Morimoto medical clinic; HIROSE CLINIC; Division of Nephrology and Kidney Center,Kobe University Graduate School of Medicine; Hattori Hospital; Meimai Central Hospital; Akashi Medical Center; Takahashi Clinic; Toume Clinic; Sakurakai Takahashi Hospital; Seiyu Clinic; Fenikkusuiwaoka Clinic; IMAI NAIKA CLINIC; Hiraoka Internal Medicine Clinic; Funamoto Clinic; Takarazuka Municipal Hospital; Hyogo College of Medicine; Hyogo Prefectural Amagasaki General Medical Center; MAKIBAYASHI CLINIC; Kitatsuji clinic; Seimei-kai Ikeda Hospital; Itami City Hospital; Kawase Clinic; Toyooka Hospital; TSUJII HAYASHI NAIKA; IHI Harima Hospital; Sano Naika Heart Clinic; Soyokaze Shinryojo; Hamano Clinic; Takasago Municipal Hospital; Ueda Internal medicine clinic (Tatsuno-city); Ueda Internal medicine clinic (Ibo-gun); Kohnan Kakogawa Hospital. Nara: Yatayama clinic; Nara Medical University; Nishimoto Medical Clinic; Kawamoto Clinic; Wada Medical Clinic; Tabata Clinic; Yamatotakada Municipal Hospital; Nara Prefecture General Medical Center; Yoshikawa Clinic; Nara Prefectural Seiwa Medical Center; Enomoto Clinic; Medical corporation iioka .Nozomi clinic. Wakayama: Shingu Municipal medical Center; Wakayama Rousai Hospital Internal Medicine; Wakayama Rousai Hospital Cardiovascular Medicine; Arida Municipal Hospital; Saiseikai Wakayama Hospital; maro clinic; Cherry-hill Hospital; Kodama Hospital; Kinokawa Clinic; KisenKD Clinic; Japanese Red Cross Wakayama Medical Center. Kyoto: Kyoto Station Takeda Dialysis Clinic; Japanese Red Cross Kyoto Daiichi Hospital; University Hospital, Kyoto Prefectural University of Medicine; Nishijin Hospital; Takeda General Hospital (Kyoto City); Ujitakeda hospital; Uji Hospital; Miyazu Takeda Hospital; KANAITSUKA CLINIC; Maizuru Kyosai Hospital; Tango Central Hospital; Kageyama clinic; Sohma Hospital; Fukuchiyama City Hospital; ICHIDA CLINIC; North Medical Center Kyoto Prefectural University Medicine; Mitsubishi Kyoto Hospital; Yamada Clinic; TOMII NAIKA IIN. Shiga: Saiseikai Shigaken Hospital; Imadu Hospital; Yujinkai Medical Corporation Yujin Yamazaki Hospital; Hikone Municipal Hospital; Omihachiman Community Medical Center; Otsu City Hospital; Toyosato Hospital; Shiga University of Medical Science Hospital; Iwamoto Orthopedic Surgery Medical Corporation; Ohashi Nephrology and Dialysis Clinic; Takashima municipal hospital. Hiroshima: Shirataki Clinic; Kimura Clinic; Hasegawa Clinic; Hiroshima Prefectural Hospital; Ichiyokai Harada Hospital; JA Hiroshima General Hospital; Shimada Family Clinic; Kure Kyosai Hospital; Medical Corporation Seikokai Tanimoto Clinic; Shobara Red Cross Hospital; Nakashima clinic; Tanimoto medical clinic; Kamikawa clinic; Onabe Surgical Hospital; Hiroshima City Asa Citizens Hospital; Kisaka Hospital; Hashimoto Kidney Clinic; Innoshima Ishikai Hospital; Nippon Kokan Fukuyama Hospital; Shonankai Association Numakuma Hospital; Ogawa Naika Ichoka Clinic; Shinjukai Takasu Clinic; Saikijin Clinic; Kobayashi Hospital. Okayama: Seiseikai Saidaiji Chuo Hospital; Okayama Medical Center; Okayama Saiseikai General Hospital; Okayama University Hospital; SATOU MEMORIAL HOSPITAL; Kasaoka Daiichi Hospital. Yamaguchi: Konan St.Hill Hospital; Morita Hospital; Ubejinshinkai hospital; Minamizono Clinic; St.Hill Hospital; SHIMONOSEKI CITY HOSPITAL; ITO NEPHRO CLINIC; fujimoto clinic; Okada Hospital; Nagato General Hospital; HOUEIKAI HOSOE CLINIC; SHIMONOSEKI MEDICAL CENTER; Iwakuni central hospital; UNO clinic hospital; Shuto General Hospital. Tottori: Sanin Rosai Hospital; Tottori University Hospital; Yoshino Miyake Station Clinic; Omodani Internal Medicine and Cardiology Clinic; ebi clinic. Shimane: Shimane University Hospital; Matsue Red Cross Hospital. Kagawa: Kagawa Rosai Hospital; Kagawa Inoshita Hospital; Mitoyo internal medicine niere clinic; Hananomiya Clinic; Takamatsu Red Cross Hospital Urology; Kagawa Prefectural Central Hospital; Kagawa University Hospital; KenAiKai medical corporation Akiyama Clinic; Medical Corporation Jujinkai Tada Clinic; Kaisei Hospital; Mizobuchi Internal Medicine Cardiology Clinic; Hasegawa Outpatients clinic for Cardiovascular Disease; Fujita Neurosurgical Clinic; Yokoi Medical Clinic; Hamamoto Clinic. Tokushima: Tamaki Aozora Hospital; Kawashima Hospital; Yoshinogawa Medical Center; Oji Paper Company Tomioka Clinic; sakura clinic; Tokushima University Hospital; Tokushima Prefectural Central Hospital; Komatsu Hinyokika; Tokushima Prefectural Miyoshi Hospital. Ehime: Matsuyama Red Cross Hospital; Ehime Prefectural Central Hospital; Matsuyama shimin Hospital; Shikoku Central Hospital of the Mutual Aid Association of Public School Teachers; Saiseikai Imabari Hospital; Saijo Central Hospital; Housyasen daiiti Hospital; Ehime University Hospital. Kochi: Kamei Clinic; Clinic Hiroto; Koryo Hospital; Kitajima Hospital; Takeshita Hospital; Minamigaoka POLARIS CLINIC; Kochi Takasu Hospital; Kochi Memorial Hospital. Fukuoka: National Hospital Organization Kyushu Medical Center; Yagi hospital; Kyushu University Hospital; Harasanshin Hospital; Japanese Red Cross Fukuoka Hospital; Syohshinkai Mito Hospital; Kuma Clinic; Kaizuka Hospital; Fukuoka City Hospital; Wakasugi Hospital; Murakami cardiovascular clinic; Koga Central Hospital; Higuchi hospital; FUKUOKA TOKUSHUKAI MEDICAL CENTER; SUENAGA CLINIC; Nagao Hospital; Fukuoka University Hospital; Medical corporation Jiryokan; Kyushu Central Hospital of the Mutual Aid Association of Public School Teachers; Division of Nephrology, Department of Medicine, Kurume University School of Medicine; OMUTA CITY HOSPITAL; St.Mary's Hospital; IMADACHI CLINIC; Kurume General Hospital; Yame General Hospital; Hamayu-kai Shinoji Hospital; Department of Kidney Center, University Hospital of Occupational and Environmental Health; Takahashi Naika Clinic; Aso Iizuka Hospital; The First Department of Internal Medicine, School of Medicine,University of Occupational and Environmental Health; Hyakutake Clinic; WATANABE CLINIC; Yoko Clinic; Nakama Municipal Hospital; Masuda-medical clinic; Matsushima Clinic; Kokura Memorial Hospital; TOKUHARA MEDICAL CLINIC; Obase Hospital; Federation of National Public Server Personnel Mutual Aid Associations SHINKOKURA Hospital; MOJI SEAMEN'S HOSPITAL; Shinyukuhashi hospital. Nagasaki: Shinagawa Surgical Hospital; Wajinkai Hospital; Nijigaoka hospital; Kouseikai Hospital; Nagasaki University Hospital; Omura Municipal Hospital; Department of Nephrology and Urology, Maeda clinic; Sasebo Chuo Hospital; National Hospital Organization Nagasaki Medical Center; Sasebo Kyosai Hospital; Japan Community Health Care Organization Isahaya General Hospital; Sasebo City General Hospital. Oita: Kasagi urology clinic; Saganoseki hospital; National Hospital Organization Beppu Medical Center; Abe Clinic; Oita University Hospital; Oita Nakamura Hospital; Tsukagawa Daiichi Hospital; Tomiku K. clinic; Shinseikai Fukushima Hospital; Oita Memorial Hospital. Saga: Saga-Ken Medical Centre Koseikan; Iwamoto Medical Hospital; Naitoh clinic. Kumamoto: JAPANESE RED CROSS KUMAMOTO HOSPITAL; Ikeda clinic (Kumamoto City); SAISEIKAI KUMAMOTO HOSPITAL; UEMURA Medical Clinic; Suefuji Clinic; Jinseikai Clinic Ozu; Akebono clinic; TsuTsumi Hospital; Japan Community Health care Organization Kumamoto General Hospital; Nanaura terasaki clinic; UTO central clinic; Higashi Diabetes and Cardiovascular Clinic. Kagoshima: Ikeda clinic (Ibusuki City); Kojukai Yotsueda Internal Medicine Clinic; UEMURA Hospital; Tanegashima medical center; IZUMI GENERAL MEDICAL CENTER; Ikeda Hospital; Tamai Clinic. Miyazaki: Tamaki Clinic; Omori Medical Clinic; Kinoshita clinic of internal medicine; Yokota Naika; Kuroki Medical Clinic; Ikei Hospital; Komidori Internal Medicine Clinic. Okinawa: Shuri jokamachi clinic Daiichi; Mamenoki Clinic; Life Care Clinic NAGAHAMA; SUNAGAWA MEDICAL CLINIC; KAWANENAIKAGEKA; Yonabaru-chuo-hospital; University Hospital of the Ryukyus; Tokuyama clinic; YOSHI-CLINIC; MATSUO TC CLINIC.
